# The Association of Micro‐ and Macro‐Nutrient Patterns With the Risk of Rheumatoid Arthritis in Newly Diagnosed Cases: A Case‐Control Study

**DOI:** 10.1002/hsr2.72553

**Published:** 2026-07-15

**Authors:** Sajedeh Jandari, Mohammad Reza Shadmand Foumani Moghadam, Mostafa Shahraki Jazinaki, Arezoo Rastegarmoghadam‐Ebrahimian, Mohammad Amin Senobari, Kazem Eslami, Mohammad Hassan Jokar, Reza Rezvani

**Affiliations:** ^1^ Department of Nutrition Sciences Mashhad University of Medical Sciences Mashhad Iran; ^2^ Service of Clinical Nutrition and Dietitian, Emam Reza Hospital Mashhad University of Medical Sciences Mashhad Iran; ^3^ Health Technology Incubator Center, Bu‐Ali Research Institute Mashhad University of Medical Science Mashhad Iran; ^4^ Department of Nutrition Varastegan Institute for Medical Sciences Mashhad Iran; ^5^ Rheumatic Diseases Research Center, School of Medicine Mashhad University of Medical Sciences Mashhad Iran

**Keywords:** case‐control, macronutrients, micronutrients, nutrient pattern, rheumatoid arthritis

## Abstract

**Background and Aims:**

This study aims to explore the nutrient patterns of macronutrients and micronutrients in healthy people and individuals newly diagnosed with rheumatoid arthritis (RA) and their associations with the risk of RA.

**Methods:**

This case‐control study involved 50 RA patients and 100 matched healthy controls. Dietary intakes were obtained using valid food frequency questionnaires (FFQ). Factor analysis was performed to identify nutritional patterns. Multivariable analysis was performed to assess the relationship between each detected nutrient pattern and the risk of RA.

**Results:**

Weight, BMI, waist circumference, physical inactivity, and prevalence of individuals with a familial history of RA were significantly higher in RA patients than in individuals without RA (*p* < 0.05). Four nutrient patterns were identified, accounting for 71.65% of the total variance. The first (high intake of vitamin D, protein, selenium, and cholesterol), third (high intake of vitamin C, calcium, and dietary fiber), and fourth (high intake of iron and vitamin E) identified patterns were inversely associated with the risk of RA, in contrast to the second pattern (high intake of total fat, trans fatty acids, saturated fatty acids (SFA), higher dietary serving score (DSS) and a higher dietary inflammatory index (DII)), which was directly related to the RA risk (*p* < 0.05).

**Conclusion:**

This study revealed that dietary patterns that are high in DSS and DII scores, total fat, SFA, and trans fatty acids may be related to the risk of RA. Also, it was shown that dietary contents of protein, cholesterol, fiber, vitamin D, vitamin C, vitamin E, selenium, and calcium could be associated with the RA pathogenesis. To reach a definitive finding regarding the association of each nutrient with the risk of RA, conducting more high‐quality cohort studies with a large sample size is needed.

## 1 Introduction

Rheumatic diseases represent a significant category of chronic non‐communicable diseases that primarily affect the musculoskeletal and joint systems [1]. Among these, rheumatoid arthritis (RA) stands out as a leading autoimmune condition characterized by progressive systemic inflammation, which not only leads to joint damage but also manifests as joint pain, swelling, and stiffness [2, 3]. The pathophysiology of RA is intricate, involving a confluence of genetic, environmental, and immunological factors that collectively disrupt normal immune function and foster chronic inflammation [4]. Globally, RA poses a considerable public health challenge, with an estimated prevalence of 0.5%–1% among the adult population, notably affecting women disproportionately compared to men [5, 6]. The onset of RA typically occurs between the ages of 40 and 60; however, it can manifest at any age, significantly impairing individuals' quality of life and imposing substantial economic burdens on healthcare systems [7, 8].

Although there is a growing body of literature on RA, its etiology remains complex and multifaceted, highlighting the roles of various genetic predispositions and environmental triggers in disease development. Traditional treatment approaches often include disease‐modifying antirheumatic drugs (DMARDs) and nonsteroidal anti‐inflammatory drugs (NSAIDs) [9, 10]. Recently, there has been an increasing interest in the potential influence of dietary factors on the inflammatory processes associated with RA. Nutritional therapy has emerged as a complementary approach to conventional treatment, aimed at alleviating symptoms, enhancing joint function, and improving overall well‐being [9]. Numerous studies have investigated specific dietary modifications and nutritional supplements for their anti‐inflammatory effects [11, 12, 13].

Investigating the impact of individual dietary components poses challenges due to the inherent complexities of nutrient interactions and potential recall biases among participants, complicating the establishment of direct correlations between specific dietary factors and RA risk [14, 15]. In contrast, focusing on patterns of macronutrients and micronutrients may offer a more nuanced and therapeutically relevant method for assessing dietary influences on RA. Micronutrients, encompassing essential vitamins and minerals, are critical to immune function, inflammatory regulation, and oxidative stress management—all key contributors to RA pathophysiology [15, 16, 17, 18]. For instance, deficiencies in certain vitamins and minerals have been associated with an increased disease activity, and inflammatory/oxidative stress status in RA [19, 20]. Additionally, macronutrients—comprising proteins, fats, and carbohydrates—may modulate immune responses and inflammatory pathways, thereby influencing the development and progression of RA [14, 21].

In light of the current challenges within this field, our research uniquely focuses on analyzing the patterns of macro‐ and micronutrients in individuals newly diagnosed with RA. By investigating these patterns, we aim to elucidate the complex network of interactions that may contribute to the risk of RA.

## 2 Methods

### 2.1 Study Design and Participants

The current case‐control research included 100 healthy persons and 50 RA patients (out of about 500 subjects) who visited the Rheumatology Clinic in Mashhad between January 2018 to February 2020. The patients included Iranian individuals with RA who had recently been diagnosed by a rheumatologist and were chosen using a simple random sample procedure. The controls were people with no inflammatory conditions, as well as joint or connective tissue illnesses, including RA. Cases and controls were matched based on age (± 5 years) and gender. The current study was conducted in compliance with the Helsinki Declaration following approval by the local Ethics Committee at Mashhad University of Medical Sciences in Mashhad, Iran (ID: IR.MUMS.MEDICAL.REC.1401.630). All participants were given verbal and written descriptions of the study's aims and procedures before providing their informed consent. More details of the study can be found in previous studies [22, 23].

### 2.2 Inclusion and Exclusion Criteria

For this study, participants were selected from individuals aged older than 18 without any connective, joint tissue diseases, or any inflammatory conditions. Those with new RA diagnoses made in the past 6 months, according to the 1987 American Rheumatism Association's updated RA classification criteria [24], were included in the case group. Eligible controls were matched in age (± 5 years) and gender following similar criteria as the case group, but without an RA diagnosis.

Participants who used certain medications affecting their nutritional status, aside from anti‐inflammatory drugs or alcohol, and those with pre‐existing conditions such as other inflammatory diseases, cardiovascular conditions such as myocardial infarction, stroke, etc., endocrine disorders like diabetes, or any disease or medication that affects their daily lifestyle and nutrition intake, and adherence to specific diets, such as diets aimed at weight gain, very‐low‐calorie diets, ketogenic diets, or vegetarian diets in the year leading up to the study, to minimize variability in dietary intake among participants and reduce extraneous variation that could obscure the dietary “signal” (i.e., the actual dietary impact related to RA), were excluded from the study.

### 2.3 Dietary Intake Assessment

Participants' typical dietary consumption over the previous year was assessed through a validated 168‐item semi‐quantitative food frequency questionnaire (FFQ) [25]. The validity and reliability of this FFQ for identifying food group consumption within the Iranian adult population were confirmed in previous studies [26]. Individuals were instructed to input their intake frequency for each food item listed in the FFQ—options included daily, weekly, monthly, or yearly—based on the item's standard serving size as indicated in the questionnaire. This allowed the calculation of the average daily consumption of calories, nutrients, and various food groups. Participants who reported a daily caloric intake of less than 800 Kcal or more than 4200 Kcal, or those who indicated they had not consumed over 40% of the food items listed in the FFQ in the year before the interview, were excluded from the final data evaluation. Subsequent analysis of the dietary information collected from the FFQ was performed using the Nutritionist IV software package (First Databank Inc., Hearst Corp.).

To identify key nutrient patterns, 13 nutrients that played an important role or had a higher correlation with the disease, along with the energy‐adjusted dietary inflammatory index (DII) and dietary serving score (DSS based on their primary ingredients or their use in cooking, were included in factor analysis [27]. DII was calculated as described in our previous study [23]. The DSS scoring system evaluated six major food groups, with a maximum score of 20 as the best. Each vegetable, fruit, and dairy group received a maximum of 4 points for every two recommended servings. A score of 4 points was assigned for consuming the recommended 4 servings of cereals/roots. For both plant and animal protein sources, 2 points were awarded for each recommended serving. The Iranian National Food Pyramid, established by the Iranian Ministry of Health, was utilized to determine recommended serving sizes for each major food group. All food groups consumed by participants were evaluated and scored based on a 2000 kcal diet [28].

### 2.4 Physical Activity Assessment

To consider the profound influence of physical activity on health, the International Physical Activity Questionnaire‐Short Form (IPAQ‐SF), a validated self‐report instrument, was utilized to evaluate the physical activity levels of each individual [29]. This questionnaire includes four broad questions designed to gather information on the length and frequency of physical activity undertaken by individuals across a range of intensities, from sedentary to vigorous, during the previous week. The total amount of physical activity was subsequently calculated in terms of metabolic equivalent task hours per week (MET‐h/week).

### 2.5 Anthropometric Measurements

Participants were asked to wear light clothing and remove shoes before being weighed with a Seca 831 digital scale accurate to 100 grams. To measure their heights in standing posture, a wall‐mounted measuring tape with a precision of 0.5 cm was used. Participants' weights in kg were divided by the square of height to calculate the Body Mass Index (BMI). Waist circumference (WC) was also measured at the narrowest circumference between the iliac crest and the rib cage.

### 2.6 Statistical Analysis

Statistical analyses were conducted using SPSS software, version 24. Qualitative and quantitative variables are presented as frequencies (percentages) and mean ± standard deviation (SD), respectively. The independent *t*‐test and the chi‐squared test were used for comparing quantitative and qualitative variables between case and control groups, respectively. To derive nutrient patterns from 15 factors, factor analysis was applied. The analysis required a minimum of 54 participants, adhering to the guideline that suggests needing at least three participants per variable for dietary pattern analysis [30]. The Kaiser–Meyer–Olkin (KMO) measure and Bartlett's test of sphericity were utilized to assess the suitability of factor analysis for the correlation among the primary food groups. Dietary patterns to be retained were selected based on an eigenvalue greater than one and the inspection of a scree plot. For easier interpretation of these patterns, a varimax rotation was applied to the factor loading matrix. Dietary pattern scores were then calculated by adding the intake of all food groups, each weighted by its respective rotated factor loading.

To compare the variables across the tertiles of dietary patterns, a chi‐square test was applied to categorical variables and ANOVA for continuous ones, with Tukey's test for post‐hoc pairwise comparisons. The risk of RA across dietary patterns was evaluated using binary logistic regression and presented as an odds ratio (OR) with 95% confidence interval (CI). In addition, multivariable analysis was performed to assess these associations while adjusting for cofounders' effects. Sample size was determined based on two complementary considerations. First, for performing factor analysis, we followed the commonly used subject‐to‐variable ratio of 10:1, such that with 15 observed variables, the minimum sample required for stable factor extraction was set to 150 participants [31]. Second, enrolling a minimum of 100 participants [32]. Therefore, the study was designed as a case–control comparison with a control: case allocation ratio of 2:1, resulting in 50 cases and 100 controls (total *N* = 150). However, the adequacy of the sample size was interpreted based on the KMO measures [33]. If the KMO is greater than 0.6, it is considered adequate for the sample size [34]. All performed analyses were considered two‐tailed, and a *p*‐value of less than 0.05 was identified as statistically significant.

## 3 Results

### 3.1 Participants' Characteristics

The mean age and BMI of individuals with and without RA were 41.70 years and 26.02 kg/m^2^, and 41.67 years and 23.89 kg/m^2^, respectively. Weight, BMI, WC, sitting and sleeping duration, and the prevalence of smoking and a family history of RA were significantly higher in patients with RA than in the control group. However, no significant difference was detected in other variables, as shown in Tables 1 and 2. The study population has been previously described in detail in our previous studies [22, 33].

**Table 1:** Comparison of quantitative characteristics between case and control groups.

Variable	Case group (*n* = 50)	Control group (*n* = 100)	*p* value^a^
Age (years)	41.70 ± 10.29	41.67 ± 10.70	0.99
Weight (kg)	67.41 ± 10.03	62.10 ± 8.56	**< 0.001**
BMI (kg/m²)	26.02 ± 3.47	23.89 ± 2.81	**< 0.001**
WC (cm)	87.93 ± 12.41	83.51 ± 7.46	**0.007**
Physical Activity (MET.h/d)	4.52 ± 2.58	6.71 ± 3.98	**< 0.001**
Sleep Duration (hours/day)	7.72 ± 1.76	7.03 ± 1.33	**0.01**
Sitting Time (hours/day)	5.96 ± 2.56	5.01 ± 1.75	**0.008**

*Note:* Bold indicates statistically significant (*p* < 0.05).

Abbreviations: BMI, body mass index; WC, Waist Circumference.

^a^ Comparison between groups was conducted by performing an independent *t*‐test.

**Table 2:** Comparison of qualitative characteristics between the case and control groups.

Qualitative variables	Variable levels	Case group (*n* = 50)	Control group (*n* = 100)	*p* value^a^
Gender	Female	44 (88)	88 (88)	1
	Male	6 (12)	12 (12)	
University Education	Yes	19 (38)	28 (28)	0.22
	No	31 (62)	72 (72)	
Smoking	Yes	17 (34)	15 (15)	**0.007**
	No	33 (66)	85 (85)	
Dietary Supplements	Yes	20 (40)	45 (45)	0.52
	No	30 (60)	55 (55)	
Family History of Arthritis	Yes	20 (40)	20 (20)	**0.009**
	No	30 (60)	80 (80)	

*Note:* Bold indicates statistically significant (*p* < 0.05).

^a^ Comparisons between groups were conducted using the Chi‐squared test.

### 3.2 Extracted Nutrient Patterns and Their Factor Loadings

Based on the factor analysis, four nutrient patterns were extracted with eigenvalues greater than one. All the extracted dietary patterns accounted for 71.65% of the total variance in food groups. The content of the extracted nutrient patterns included:

1. First Nutrient Pattern: High intake of vitamin D, protein, selenium, and cholesterol.
2. Second Nutrient Pattern: High intake of total fat, trans fatty acids, saturated fatty acids (SFA), and higher DII and DSS scores.
3. Third Nutrient Pattern: High intake of vitamin C, calcium, and dietary fiber.
4. Fourth Nutrient Pattern: High intake of iron and vitamin E.

Table 3 lists the factor loadings for both micronutrient and macronutrient groups in the prevalent dietary patterns. The factor loadings less than 0.30 are not presented in this table. Therefore, the higher the factor loading of any group of micronutrients in a particular pattern or factor, the greater its contribution to that pattern of micronutrients. The KMO index (0.658) and Bartlett's test of sphericity (*p* < 0.001) validated the adequacy of sample size and intercorrelation among variables for strong factor analysis.

**Table 3:** Factor loadings of micronutrients and macronutrients in major dietary patterns.

Food groups	Dietary patterns			
	1	2	3	4
Protein per 1000 kcal	0.852			
Fat per 1000 kcal		0.807		
Carbohydrate per 1000 kcal	−0.546	−0.722		
Dietary Fiber per 1000 kcal			0.706	
Saturated Fatty Acids per 1000 kcal		0.669		
Trans Fatty Acids per 1000 kcal		0.467		
Cholesterol per 1000 kcal	0.861			
Calcium per 1000 kcal			0.658	−0.453
Iron per 1000 kcal				0.842
Vitamin E per 1000 kcal				0.795
Vitamin C per 1000 kcal			0.785	
Vitamin D per 1000 kcal	0.826			
Selenium per 1000 kcal	0.848			
Energy‐adjusted DII using density method		0.484	−0.664	
Dietary serving score from 20	−0.684	0.722		

*Note:* Dietary patterns were identified by performing factor analysis.

### 3.3 Significance Levels of Different Variables in Nutrient Patterns

The analysis demonstrated that age in the fourth nutrient pattern, family history of RA, BMI, WC, and sitting time in the first micronutrient pattern, and age and smoking in the third micronutrient pattern showed statistically significant differences among the tertiles of these patterns and were considered confounding factors. Nevertheless, no statistically significant differences were seen for gender, occupation, education, height, physical activity, and sleep duration between the tertiles of any detected nutrient patterns (Table 4).

**Table 4:** Significance levels of different variables in nutrient patterns.

Variables	Dietary patterns			
	1	2	3	4
Age	0.91	0.537	**0.047**	**< 0.001**
Gender	0.346	0.19	0.629	0.422
Occupation	0.238	0.698	0.058	0.305
Education	0.668	0.42	0.086	0.318
Family History of Rheumatoid Arthritis	**0.022**	0.348	0.523	0.642
Smoking	0.127	0.47	**0.008**	0.181
Weight (kg)	0.146	0.263	0.826	0.144
Height (cm)	0.215	0.967	0.967	0.319
BMI	**0.022**	0.126	0.752	0.192
WC	**0.007**	0.961	0.534	0.098
Physical Activity (h/week)	0.49	0.377	0.963	0.43
Sitting Time	**0.04**	0.195	0.886	0.24
Sleep Duration	0.576	0.050	0.712	0.531

*Note:* A one‐way ANOVA and chi‐square tests were performed to compare the quantitative and qualitative variables between the tertiles of each dietary pattern. Bolds indicate statistically significant (*p* < 0.05).

Abbreviations: BMI, body mass index; WC, waist circumference.

1. Pattern 1: High intake of vitamin D, protein, selenium, and cholesterol. 2. Pattern 2: High intake of total fat, trans fatty acids, saturated fatty acids (SFA), and higher DII and DDS scores. 3. Pattern 3: Third Nutrient Pattern: High intake of vitamin C, calcium, and dietary fiber. 4. Pattern 4: Fourth Nutrient Pattern: High intake of iron and vitamin E.

### 3.4 Comparison of Macronutrients and Micronutrients Among Tertiles of Different Patterns and Healthy and Diseased Individuals

**Figure 1:**
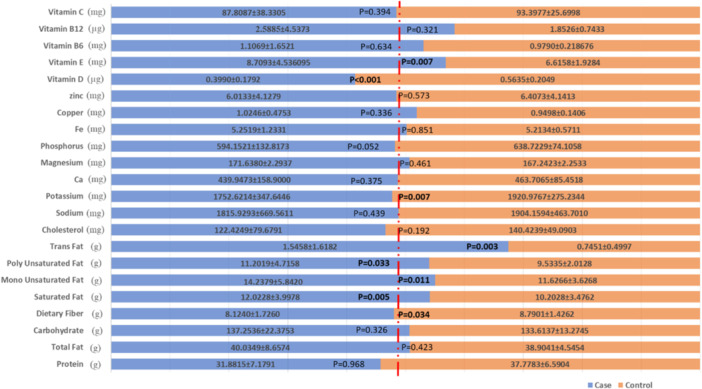
Comparison of nutrient intake per day between rheumatoid arthritis patients and the control group.

The average intakes of some main nutrients were statistically different between healthy and RA groups, where dietary intake of fiber, potassium, and vitamin D was significantly higher in individuals without RA than in the cases, and intake of trans fatty acids, MUFA, PUFA, SFA, and vitamin E was significantly higher in RA patients compared to the control group. Other results are summarized in Figure 1. As was shown in Table 5, a significant difference in intakes of protein, carbohydrate, trans fatty acid, cholesterol, calcium, vitamin D, fruits, white meat, and dairy was observed in the tertiles of the first pattern (*p* < 0.05). Also, there was a significant difference in the consumption of protein, carbohydrate, fat, fiber, SFA, trans fatty acid, calcium, vitamin D, vitamin E, vegetables, fruits, white meat, and dairy across the tertiles of the second dietary pattern. Furthermore, a significant difference in total energy, protein, fat, fiber, SFA, trans fatty acid, calcium, iron, vitamin C, vegetables, fruit, red meat, fast food, dairy, rice, and bread was detected among tertiles of the third pattern. In addition, there was a significant difference in carbohydrate, fat, fiber, calcium, iron, cholesterol, vitamin E, vitamin C, fruits, vegetables, white meat, and dairy across the tertiles of the fourth pattern.

**Table 5:** Comparison of mean dietary, macronutrient, and micronutrient intakes per day between tertiles of nutrient patterns.

Diet parameters	Pattern 1			Pattern 2			Pattern 3			Pattern 4		
	T1	T2	T3	T1	T2	T3	T1	T2	T3	T1	T2	T3
**Energy (kcal)**	2298.66 ± 717.42	2166.92 ± 446.23	2210.97 ± 392.24	2194.11 ± 594.46	2235.21 ± 373.86	2247.23 ± 618.14	2428.76 ± 653.49**	2284.58 ± 355.24**	1963.21 ± 455.71**	2166.18 ± 649.5	2247.83 ± 288.01	2239.25 ± 664.08
**Protein (g)**	30.1 ± 4.46**	34.85 ± 3.54**	42.55 ± 7.03**	35.04 ± 7.33*	37.99 ± 6.34*	34.47 ± 7.82*	33.26 ± 8.09*	37.14 ± 7.06*	37.1 ± 6.09*	36.36 ± 9.16	36.08 ± 4.45	32.97 ± 5.69
**Carbohydrate (g)**	141.7 ± 21.67**	136.92 ± 11.34**	127.35 ± 12.95**	149.99 ± 17**	131.78 ± 10.81**	124.19 ± 10.5**	133.63 ± 21.59	136.55 ± 14.17	135.78 ± 14.25	137.62 ± 17.44*	132.29 ± 10.42*	128.49 ± 13.23*
**Fat (g)**	38.89 ± 8.41	38.93 ± 4.86	39.39 ± 4.72	33.3 ± 5.04**	39.67 ± 2.88**	44.24 ± 4.64**	40.82 ± 7.79*	37.94 ± 4.61*	38.45 ± 5.48*	37.84 ± 5.94**	40.32 ± 4.12**	43.29 ± 5.35**
**Fiber (g)**	8.97 ± 2.06	8.51 ± 1.19	8.27 ± 1.2	9.3 ± 1.61**	8.64 ± 1.24**	7.81 ± 1.44**	7.34 ± 1.26**	8.85 ± 0.86**	9.56 ± 1.55**	7.93 ± 1.19**	8.7 ± 1.1**	9.17 ± 1.82**
**Saturated Fatty Acid (g)**	11.69 ± 4.31	10.84 ± 3.33	10.05 ± 3.47	8.79 ± 2.81**	9.84 ± 2**	13.95 ± 4**	11.4 ± 4.23*	9.75 ± 3.4*	11.43 ± 3.43*	10.42 ± 3.28	10.37 ± 2.55	10.43 ± 3.07
**Trans Fatty Acid (g)**	1.35 ± 1.56*	0.8 ± 0.52*	0.86 ± 0.8*	0.65 ± 0.54**	0.83 ± 0.63**	1.52 ± 1.55**	1.55 ± 1.54**	0.79 ± 0.53**	0.66 ± 0.63**	0.71 ± 0.42	0.76 ± 0.42	0.81 ± 0.7
**Cholesterol (mg)**	83.64 ± 22.14**	123.14 ± 20.82**	193.52 ± 65.35**	126.53 ± 53.83	142.75 ± 53.13	131.03 ± 75.19	136 ± 80.79	137.29 ± 44.1	127.01 ± 54.6	122.38 ± 38.93*	134.23 ± 34.68*	104.95 ± 43.6*
**Calcium (mg)**	413.5 ± 99.96**	466.23 ± 116.45**	491.31 ± 116.22**	424.53 ± 94.02*	471.31 ± 79.23*	475.2 ± 153.42*	375.85 ± 81.28**	442.74 ± 55.09**	552.45 ± 120.05**	528.08 ± 160.88*	477.7 ± 69.4*	427.61 ± 109.07*
**Iron (mg)**	5.15 ± 0.7	5.22 ± 0.78	5.26 ± 1.04	5.24 ± 0.67	5.23 ± 0.62	5.17 ± 1.16	4.97 ± 0.95*	5.26 ± 0.56*	5.41 ± 0.93*	4.44 ± 0.75**	5.1 ± 0.3**	5.82 ± 0.77**
**Vitamin E (mg)**	7.42 ± 3.13	7.53 ± 3.26	6.81 ± 3.14	6.17 ± 1.73*	7.13 ± 2.72*	8.45 ± 4.18*	7.31 ± 4.19	7.21 ± 2.74	7.23 ± 2.35	5.28 ± 1.29**	7.06 ± 1.28**	10.62 ± 3.94**
**Vitamin C (mg)**	97.61 ± 41.69	86.15 ± 21.37	90.16 ± 23.81	98.18 ± 36.47	92.15 ± 23.81	83.59 ± 28.7	66.03 ± 17.34**	92.61 ± 16.86**	115.28 ± 31.71**	84.8 ± 28.82*	91.5 ± 17.25*	106.95 ± 33.56*
**Vitamin D (µg)**	0.34 ± 0.12**	0.49 ± 0.11**	0.68 ± 0.21**	0.52 ± 0.23*	0.55 ± 0.21*	0.44 ± 0.16*	0.46 ± 0.26	0.55 ± 0.17	0.50 ± 0.18	0.47 ± 0.19	0.54 ± 0.14	0.49 ± 0.27
**Vegetables (g)**	375.42 ± 201.81	372.85 ± 140.65	407.82 ± 148.57	368.03 ± 166.6*	443.14 ± 126.47*	343.63 ± 185.47**	303.14 ± 130.45**	422.5 ± 133.12**	428.36 ± 196.09**	345.81 ± 171.93*	436.06 ± 138.97*	465.85 ± 183.91*
**Fruits (g)**	706.3 ± 403.74*	587.12 ± 206.94*	585.66 ± 166.83*	734.35 ± 397.38**	599.08 ± 142.82**	545.64 ± 214.09**	509.19 ± 202.59**	702.02 ± 249.07**	667.87 ± 344.62**	552.47 ± 224.53*	610.89 ± 179.9*	724.7 ± 379.38*
**Red Meat (g)**	32.38 ± 28.2	25.82 ± 16.29	26.63 ± 21.1	26.29 ± 22.33	25.47 ± 17.94	33.07 ± 26	34.19 ± 26.16*	28.08 ± 21*	22.56 ± 18.37*	21.14 ± 13.81	26.7 ± 16.18	27.43 ± 24.5
**White Meat (g)**	31.68 ± 21.01**	61.02 ± 33.33**	115.2 ± 65.31**	69.33 ± 56.03**	89.04 ± 57.7**	49.53 ± 47.07**	66.48 ± 67.82	83.98 ± 55.3	57.44 ± 38.11	71.56 ± 57.44*	71.31 ± 39.34*	44.88 ± 37.76*
**Fast Food (g)**	7.98 ± 24.02	4.62 ± 9.61	2.42 ± 2.47	2.79 ± 3.33	3.21 ± 4.97	9.01 ± 25.12	10.15 ± 25.15*	3.03 ± 3.23*	1.83 ± 2.66*	4.6 ± 11.64	4.07 ± 7.2	1.71 ± 2.84
**Dairy (g)**	375.42 ± 201.81*	372.85 ± 140.65*	407.82 ± 148.57*	263.41 ± 128.44**	355.67 ± 121.74**	372.16 ± 162.56**	265.47 ± 127.73**	325.47 ± 108.43**	398.08 ± 165.92**	443.56 ± 131.61**	369.43 ± 86.46**	257.09 ± 126.6**
**Bread (g)**	706.3 ± 403.74	587.12 ± 206.94	585.66 ± 166.83	118.02 ± 61.25	107.85 ± 64.7	92.04 ± 54.15	125.05 ± 78.71**	107.42 ± 44.99**	85.44 ± 46.85**	90.17 ± 50.99	105.67 ± 41.03	88.49 ± 52.37
**Rice (g)**	32.38 ± 28.2	25.82 ± 16.29	26.63 ± 21.1	161.17 ± 85.89	171.78 ± 90.32	132.16 ± 83.98	155.97 ± 88.03**	183.98 ± 72.9**	125.16 ± 92.83**	162.94 ± 83.83	182.03 ± 59.22	136.14 ± 108.95
**Coffee and Chocolate (g)**	31.68 ± 21.01	61.02 ± 33.33	115.2 ± 65.31	53.38 ± 87.43	69.89 ± 119.13	114.15 ± 354.97	137.44 ± 356.04	48 ± 75.38	51.98 ± 110.44	84.64 ± 165.27	63.96 ± 116.3	69.35 ± 159.13
**Tea (g)**	7.98 ± 24.02	4.62 ± 9.61	2.42 ± 2.47	670.98 ± 354.51	588.83 ± 217.86	656.66 ± 386.42	694 ± 431.99	634.93 ± 253.07	587.54 ± 264.43	629.45 ± 414.95	663.29 ± 210.21	610.57 ± 341.27
**Sugar (g)**	375.42 ± 201.81	372.85 ± 140.65	407.82 ± 148.57	23.65 ± 12.65	38.91 ± 135.87	26.88 ± 22.26	33.7 ± 23.42	19.66 ± 8.51	36.08 ± 135.89	19.71 ± 10.91	18.99 ± 10	22.83 ± 22.17

*Note:* All nutrients are reported per 1000 kcal. Energy and main food groups are reported as total intake. A one‐way ANOVA test was performed to compare variables across the tertiles of each nutrition pattern.

** *p* < 0.005

* *p* < 0.05.

### 3.5 The Association Between Risk of RA and Tertiles of Nutrient Patterns

Binary logistic regression showed a significant inverse relationship between adherence to the highest tertile of the first and third dietary patterns and odds of RA in crude models. Also, in the crude model, a significant direct association was observed between adherence to the highest tertile of the second dietary pattern and odds of RA. Multivariable analysis indicated a significant inverse association between adherence to the highest tertile of the first dietary pattern and an 89% to 92% reduction in odds of RA compared to the first tertile in all adjusted models. Also, adherence to the third dietary pattern had a significant inverse association with the 64% to 75% reduction in odds of RA compared to the first tertile in statistical models 1, 2, and 4. Also, in models 1, 2, and 3, adherence to the highest tertile of the second dietary pattern was significantly associated with a 2.20 to 6.52‐fold higher odds of RA. In addition, although no significant association was observed between adherence to the highest tertiles of the fourth pattern and risk of RA in comparison to the first tertile, but the second tertile of the fourth pattern was significantly associated with a 71% to 79% reduction in RA risk compared to the first tertile in all adjusted models (Table 6).

**Table 6:** Logistic regression results before and after adjusting for confounders to examine the association between nutrient patterns and rheumatoid arthritis.

Statistical model	Micronutrient pattern 1		Micronutrient pattern 2		Micronutrient pattern 3		Micronutrient pattern 4	
	T2	T3	T2	T3	T2	T3	T2	T3
Crude Model	0.337 (0.148‐0.767)*	0.128 (0.048‐0.339)**	0.512 (0.200‐1.313)	2.528 (1.112‐5.744)*	0.260 (0.109‐0.621)*	0.324 (0.140‐0.752)*	0.266 (0.099‐0.709)*	1.506 (0.679‐3.339)
*p*‐value	**0.010**	**< 0.001**	0.164	**0.027**	**0.002**	**0.009**	**0.008**	0.313
Model 1	0.361 (0.157‐0.833)*	0.130 (0.048‐0.346)**	0.525 (0.201‐1.369)	2.201 (1.318‐5.529)*	0.261 (0.107‐0.633)*	0.363 (0.146‐0.900)*	0.215 (0.075‐0.615)*	1.477 (0.649‐3.361)
*p*‐value	**0.017**	**< 0.001**	0.188	**0.022**	**0.003**	**0.029**	**0.004**	0.353
Model 2	0.487 (0.182‐1.305)	0.111 (0.034‐0.361)**	0.511 (0.193‐1.351)	2.524 (1.154‐6.332)*	0.256 (0.093‐0.701)*	0.250 (0.089‐0.705)*	0.292 (0.088‐0.962)*	2.617 (0.985‐6.956)
*p*‐value	0.152	**< 0.001**	0.176	**0.006**	**0.008**	**0.009**	**0.043**	0.054
Model 3	0.576 (0.193‐1.718)	0.113 (0.029‐0.437)*	0.489 (0.125‐1.912)	6.524 (1.820‐23.383)*	0.218 (0.155‐1.525)*	0.486 (0.089‐0.705)	0.254 (0.069‐0.944)*	1.537 (0.529‐4.460)
*p*‐value	0.322	**0.002**	0.304	**0.004**	**0.018**	0.216	**0.041**	0.429
Model 4	0.464 (0.173‐1.244)	0.088 (0.026‐0.302)**	0.430 (0.141‐1.315)	2.407 (0.861‐6.727)	0.358 (0.133‐0.962)*	0.333 (0.117‐0.949)*	0.292 (0.094‐0.912)*	1.829 (0.861‐4.830)
*p*‐value	0.127	**< 0.001**	0.139	0.094	**0.042**	**0.040**	**0.034**	0.223

*Note:* Bolds indicate statistical significance (*p* < 0.05).

Models:

Model 1 is Adjusted for age, gender, and dietary energy intake.

Model 2 is Adjusted for age, BMI, waist circumference, physical activity, and sleep duration.

Model 3 is Adjusted for gender, smoking, education, dietary supplements, steroid use, and family history of rheumatoid arthritis.

Model 4 is Adjusted for sleep duration, sitting time, physical activity time, and smoking.

* *p* < 0.05

** *p* < 0.005.

## 4 Discussion

In this research, we conducted a pioneering investigation into the association between micronutrient patterns and the odds of RA within the Iranian population. Our findings unveil several distinct micronutrient patterns, notably revealing that the first, third, and fourth patterns exhibit an inverse relationship with RA, while the second pattern was directly associated with an increased risk. Given that the factor analysis revealed specific dietary patterns, results should be interpreted within the framework of these patterns, while conclusions based on individual micronutrients warrant careful consideration. The first dietary pattern is characterized by a high intake of protein, vitamin D, selenium, and cholesterol, and it demonstrates an inverse relationship with the risk of RA. Numerous studies corroborate the notion that selenium [35, 36] and vitamin D intake [37, 38] are linked to reduced RA risk. A study conducted by Zamani et al. illustrated that selenium supplementation contributed to significant reductions in inflammatory markers such as erythrocyte sedimentation rate (ESR) and anti‐citrullinated protein antibodies (anti‐CCP) among patients with RA (however, although these changes were significant within the intervention group, the between‐group differences were not statistically significant) [39]. Similarly, vitamin D intake correlates with significantly decreased risk of RA, as evidenced by Merlino et al. in their prospective cohort study involving 29,368 women aged 55 to 69 years [37, 38]. Nonetheless, the mixed results in the literature underscore the complexities surrounding dietary protein's role in RA; for instance, a cohort study by Benito‐Garcia et al. involving 82,063 participants observed no definitive association between dietary protein sources and RA incidence [40].

The discrepancies in these findings may arise from methodological variations, such as dietary intake assessment techniques and population characteristics. As existing evidence delineates, vitamin D functions as an immune modulator, inhibiting the synthesis of interleukins and tumor necrosis factor‐alpha (TNF‐α), ultimately mitigating inflammation in both systemic circulation and synovial tissues [41]. Additionally, selenium's role as an antioxidant enhances glutathione peroxidase activity, further contributing to its anti‐inflammatory properties [42, 43]. Although dietary cholesterol was identified as a component of the first micronutrient pattern, available evidence suggests that the relation between dietary cholesterol intake and RA risk is complicated and could contribute to the prevention or susceptibility to the autoimmune diseases [44]. In contrast, our second identified dietary pattern, marked by high total fat, trans fatty acids, and saturated fatty acids in conjunction with higher DII and DSS scores, correlate with an increased risk of RA. In this regard, our previous study revealed that receiving diets with higher DII was significantly associated with the risk of RA [23]. Furthermore, Rezazadeh et al. reported that adherence to a Western dietary pattern that was identified by high‐fat dairy products, high‐fat meats, sweet snacks, and refined grains was significantly associated with developing RA [45]. In another study, Nezamoleslami et al. reported a significant direct association between adherence to the Western dietary pattern, and heightened RA risk [46]. Furthermore, an increase in saturated fatty acid consumption may exacerbate muscle atrophy among RA patients, as reported in prospective study [47]. Importantly, evidence suggests inflammatory diets, characterized by elevated DII scores, are linked to the enhanced presence of inflammatory biomarkers such as C‐reactive protein (CRP), significantly increasing RA susceptibility [48, 49, 50, 51]. This relationship underscores the synergistic effect of dietary factors on inflammation through the promotion of pro‐inflammatory cytokines, contributing to disease activity in RA patients [49].

Conversely, our findings revealed an association between a diet rich in dietary fiber, vitamin C, and calcium, with a decreased risk of RA. Studies corroborate the protective role of dietary fiber, with several investigations linking higher fiber intake to diminished inflammatory markers [1, 52, 53]. Additionally, Stone et al. reported a markly deficiency in dietary calcium among RA patients, with only 23% of patients meeting the recommended daily allowance (RDA) [54]. Clinical trials further bolster this notion; for instance, a supplementation study utilizing 500 mg of vitamin C combined with vitamin E has demonstrated significant improvements in RA symptoms, including pain and joint swelling [55]. Also, higher dietary fiber intake has been associated with reduced inflammatory burden of RA [53]. It is essential to recognize that low‐fiber diets may promote dysbiosis, immune dysregulation, and increased systemic inflammation, thereby exacerbating RA symptoms [1]. As fiber undergoes fermentation by gut bacteria, it generates short‐chain fatty acids (SCFAs), which have been shown to exert anti‐inflammatory effects [56]. Furthermore, higher vitamin C intake has been associated with reduced oxidative stress and may contribute to protection against oxidative stress [57].

Lastly, our study identified a significant association between a diet pattern rich in vitamin E and iron and reduced odds of RA (the second tertile of adherence to this dietary pattern compared to the first). A clear gradient pattern was observed across tertiles, with higher adherence to this pattern, as much as the highest tertile had a significantly higher intake of fats, fiber, vitamin C, vegetables, and fruits and progressively lower intakes white meat and dairy compared to the first. It has been documented that RA patients often display suboptimal intake levels of vitamin E relative to the RDA [54, 58]. Notably, a previous clinical trial suggest that combined supplementation with vitamin E and C may improve disease activity and inflammatory outcomes in patients with RA [55]. The protective nature of the identified micronutrient pattern can be attributed to the antioxidant properties of vitamin E. Research suggests that vitamin E deficiencies can invoke immune suppression and enhance inflammatory processes, thereby reducing the body's ability to regulate inflammation [59]. The association between dietary iron intake and RA remains controversial; however, a previous some study was reported no significant relationship [40], and also, in another study, it was mentioned that disturbances in iron homeostasis may contribute to the inflammatory processes observed in individuals with RA [60]. It is necessary to interpret the findings of this study as identified dietary patterns, not a single food that may exist in each detected dietary pattern.

In the present case‐control study, dietary intake of energy‐adjusted vitamin D was significantly lower in individuals with RA than in others. This finding could be linked to the immune‐modulatory role of vitamin D in the body [61]. In addition, dietary intake of vitamin E, monounsaturated fatty acids, polyunsaturated fatty acids, trans fatty acids, and saturated fatty acids that were adjusted based on the energy intakes was significantly higher in RA patients compared to the control group. In animal studies, it was shown that adherence to a high‐fat diet in rats led to an increase in inflammatory response in the hypothalamus [62]. Higher vitamin E intake in RA patients may be correlated to the higher consumption of unsaturated fatty acids in this group, which could be due to higher consumption of vitamin E and unsaturated fatty acid‐rich sources such as vegetable oils, seed oils, etc. [63]. Due to the use of secondary data, calculating the power of analysis was necessary.

Considering a significance level of *p* < 0.05 and a case‐to‐control ratio of 1:2, statistical power was defined as high when exceeding 80% and moderate when between 60% and 79% [64, 65].

Based on the available sample size, the study is likely to have adequate power to detect moderate to strong associations between dietary patterns and rheumatoid arthritis risk. In contrast, weaker associations close to the null (OR ≈ 1) may not be reliably detected. In addition, effect estimates at the extreme ends should be interpreted with caution, as they may be unstable due to the limited sample size and sparse data [55, 56].

To our best knowledge, our study was the first of its kind conducted within this demographic, emphasizing the importance of understanding the relationship between micronutrient intake patterns and the odds of RA. Moreover, including well‐matched controls and adjusting for confounders, which strengthened the findings, were other strengths of this study. However, some limitations may have influenced our findings. This study has a limitation, including the potential for recall bias due to using the FFQ questionnaire. Also, due to a lack of available data regarding sun exposure time and endogenous synthesis of vitamin D, medication duration, and the use of medications other than steroids, these factors could not be included in the analyses. Additionally, as with other case‐control studies, establishing a definitive temporal relationship and causality between dietary patterns and RA risk was not possible, including the unavailability of inflammatory markers, a small sample size, and an inability to exclude anti‐inflammatory drug use as a confounder.

## 5 Conclusion

In conclusion, this case‐control study revealed that diets with a pattern rich in vitamin D, protein, selenium, and cholesterol or rich in vitamin C, calcium, and dietary fiber could be inversely associated with RA risk. In addition, it was shown that adherence to the dietary pattern rich in total fat, trans fatty acids, and saturated fatty acids (SFA), and with higher DSS and DII scores may be directly associated with the odds of RA. To reach a definite conclusion regarding the association between dietary patterns and the risk of RA, conducting a cohort study with a large sample size is required.

## Author Contributions

**Sajedeh Jandari:** conceptualization, project administration, writing – review and editing, data curation, investigation, methodology. **Mohammad Reza Shadmand Foumani Moghadam:** formal analysis, data curation. **Mostafa Shahraki Jazinaki:** writing – review and editing. **Arezoo Rastegarmoghadam‐Ebrahimian:** Writing – original draft. **Mohammad Amin Senobari:** formal analysis, writing – original draft. **Kazem Eslami:** writing – review and editing. **Mohammad Hassan Jokar:** resources, methodology, investigation. **Reza Rezvani:** conceptualization, supervision, writing – review and editing, methodology, funding acquisition.

## Ethics Statement

This study proposal was approved by the Ethics Committee at Mashhad University of Medical Sciences in Mashhad, Iran, by following registration ID: IR. MUMS. MEDICAL. REC.1401.630.

All authors have read and approved the final version of the manuscript. Reza Rezvani had full access to all of the data in this study and takes complete responsibility for the integrity of the data and the accuracy of the data analysis. All participants signed the informed consent form before enrolling in our study.

## Conflicts of Interest

The authors declare no conflicts of interest.

## Transparency Statement

The lead author Reza Rezvani affirms that this manuscript is an honest, accurate, and transparent account of the study being reported; that no important aspects of the study have been omitted; and that any discrepancies from the study as planned (and, if relevant, registered) have been explained.

## Data Availability

The data that support the findings of this study are available from the corresponding author upon reasonable request.
